# Two Novel Neutral Cyclometalated Iridium(III) Complexes Based on 10,11,12,13-Tetrahydrodibenzo[a,c]phenazine for Efficient Red Electroluminescence

**DOI:** 10.3390/molecules28124865

**Published:** 2023-06-20

**Authors:** Yuzhen Yang, Han Zhao, Weiqiao Zhou, Qin Zeng, Zihao Zhang, Junjie Jiang, Yongyang Gong, Yanqin Miao, Song Guo, Yuanli Liu

**Affiliations:** 1 Guangxi Key Laboratory of Optical and Electronic Materials and Devices, College of Materials Science and Engineering, Guilin University of Technology, Guilin 541004, China; yangyuzhen812@163.com (Y.Y.); 1020200156@glut.edu.cn (H.Z.); wangyi123zhou@163.com (W.Z.); zqinnn@163.com (Q.Z.); 1020190104@glut.edu.cn (Z.Z.); jiejiangjjj0513@163.com (J.J.); yygong@glut.edu.cn (Y.G.); 2 MOE Key Laboratory of Interface Science and Engineering in Advanced Materials, Taiyuan University of Technology, Taiyuan 030024, China; miaoyanqin@tyut.edu.cn

**Keywords:** iridium(III) complexes, red emission, phosphorescence, OLEDs

## Abstract

Two novel neutral phosphorescent iridium(III) complexes (Ir1 and Ir2) were rationally designed and synthesized with high yields using 10,11,12,13-tetrahydrodibenzo[a,c]phenazine as the main ligand. The two complexes showed bright-red phosphorescence (625 nm for Ir1, and 620 nm for Ir2, in CH_2_Cl_2_), high-luminescence quantum efficiency (0.32 for Ir1, and 0.35 for Ir2), obvious solvatochromism and good thermostability. Then, they were used to fabricate high-efficiency red OLEDs via vacuum evaporation; the maximum current efficiency, power efficiency, and external quantum efficiency of the red devices based on Ir1 and Ir2 are 13.47/15.22 cd/A, 10.35/12.26 lm/W, and 10.08/7.48%, respectively.

## 1. Introduction

In the last two decades, OLEDs (Organic Light-Emitting Diodes), as a potential technology for solid-state lighting and displays, have attracted more and more attention in academia and industry due to their unique characteristics, such as wide-viewing angle, ultra-thin thickness, full-color displays, flexibility, and so on [1,2,3,4,5,6,7,8]. Materials, especially the emissive layer (EML) materials in OLEDs, play a vital role on the device performances. As we know, there exists 25% singlet excitons and 75% triplet excitons resulted from the electrical excitation. Transitional fluorescent materials can only utilize 25% singlet excitons, which limits the internal quantum efficiency [9,10,11,12]. In order to achieve high-efficiency devices, phosphorescent transition-metal complexes, such as iridium(III) complex and platinum(II) complex, are usually introduced into OLEDs as EML, owing to their significant properties, such as tunable phosphorescent wavelength, abundant triplet-excited states, high-luminescence quantum efficiency, large spin-orbital coupling (SOC) constant and excellent photo- and chemical stability [13,14,15,16,17]. In addition, heavy atom effect can effectively enhance the progress of intersystem crossing from excited-singlet state to excited-triplet state. For instance, in the iridium(III) complex-based OLEDs, both excited-singlet and excited-triplet state excitons can be captured simultaneously, thus obtaining 100% internal quantum efficiency theoretically [18,19,20]. Red emission is one of the important primary colors for display applications, and has aroused tremendous interest. Designing and fabricating novel, efficient, red iridium(III) complexes are of great significance and practical values [21,22].

Recently, several new red iridium(III) complexes were reported to achieve high-performance OLEDs [23,24,25,26]. For example, in 2019, Zheng et al. reported two rapid-room temperature syntheses of red iridium(III) complexes, with Ir–S–P–S structures, for efficient OLEDs with a maximum power efficiency of 44.40 lm/W and a maximum external quantum efficiency of 24.90%. Zuo et al. reported a series of iridium(III) complexes based on thienylpyridine to fabricate yellow-to-deep red OLEDs with low efficiency roll-off. Although great efforts have been devoted to designing and synthesizing new red iridium(III) complexes, the most common strategy is to enlarge the degree of conjugation or introduce donor and acceptor groups, thereby reducing the energy gap between the highest occupied molecular orbital (HOMO) and the lowest unoccupied molecular orbital (LUMO); the obtained complexes are often with poor solubility and low yield. Thus, it is necessary to explore novel, efficient, red iridium(III) complexes with appreciable yields and simple structure, and it is still an arduous assignment owing to the lacking versatility of the reported luminogens.

Herein, two high-efficient, red-neutral iridium(III) complexes (Ir1 and Ir2) using 10,11,12,13-tetrahydrodibenzo[a,c]phenazine as the main ligand, and acetylacetone and picolinic acid as auxiliary ligand, respectively, have been rationally designed and synthesized with high yield and good solubility. A fused ring, 10,11,12,13-Tetrahydrodibenzo[a,c]phenazine, can enrich the degree of conjugation of the complex, thereby leading to redshift of the emission; the saturated ring in the main ligand can increase the solubility of the complex in organic solvents. Both complexes showed excellent photophysical properties and intense red phosphorescence with high-luminescent quantum efficiency. Then, Ir1 and Ir2 were used to fabricate red OLEDs via vacuum evaporation. It is worth noting that different auxiliary ligands showed great influence on the device performances. The maximum emission peaks of the devices based on Ir1 and Ir2 were at 612 and 591 nm, respectively. Among them, the maximum brightness and maximum EQE of the Ir1-based device were 25,970 cd/m^2^ and 10.08%, respectively.

## 2. Results

### 2.1. Structural Characterization

**Figure 1:**
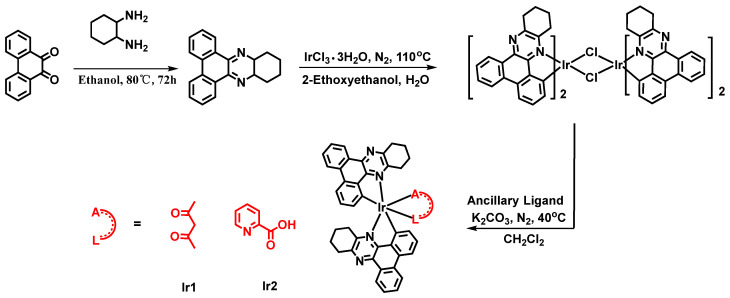
Synthetic route and chemical structures of complexes Ir1 and Ir2.

Complexes Ir1 and Ir2 were rationally designed and synthesized as illustrated in Figure 1. At first, the main ligand, 10,11,12,13-tetrahydrodibenzo[a,c]phenazine, was synthesized through a cyclization reaction between 9,10-phenanthraquinone and 1,2-diaminocyclohexane at 80 °C in ethanol, and obtained by recrystallization with high yield. Then, the cyclometalated iridium(III) dimers were prepared in the light of the reported literatures. At last, complexes Ir1 and Ir2 were obtained by the reaction of cyclometalated iridium(III) dimers with acetylacetone and picolinic acid, using anhydrous K_2_CO_3_, in a mixture of dichloromethane and methanol at room temperature, respectively. The targeted complexes Ir1 and Ir2 were separated as red powders; their chemical structures were verified completely by nuclear magnetic resonance (NMR) and mass spectra. Ir1 and Ir2 exhibited good solubility in common organic solvents, owing to the naphthenic hydrocarbon unit in the main ligand. Because of the symmetric characteristic of the auxiliary ligand acetylacetone, the two main ligands in Ir1 showed the identical resonance signals. Meanwhile, the structure of picolinic acid is asymmetric. As a result, the two main ligands in Ir2 exhibited two distinct resonance signals as illustrated in ^1^H NMR spectra (Supplementary Materials) [27]. The other resonance signals are consistent with the structures of both complexes. However, the photophysical properties of both complexes have been studied via UV/vis absorption spectrometry, and steady-state and transient phosphorescence spectrometry.

### 2.2. Photophysical Properties

**Figure 2:**
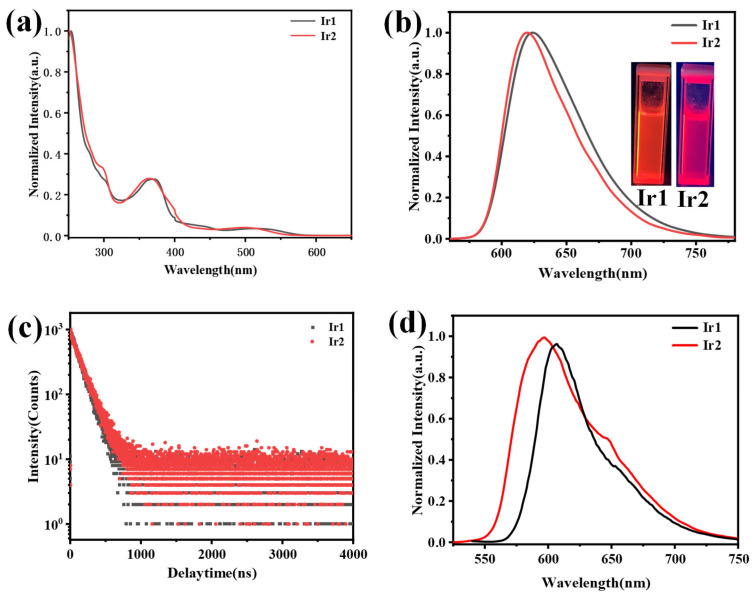
(**a**,**b**) The absorption spectra (**left**) and emission spectra (**right**) of Ir1 (black line) and Ir2 (red line) in dichloromethane, insert: the photographs of Ir1 and Ir2 in CH_2_Cl_2_ solution. (**c**) The decay curves of phosphorescent lifetime for Ir1 and Ir2 in solid states. (**d**) The low temperature emission spectra of Ir1 and Ir2 at 77 K.

**Figure 3:**
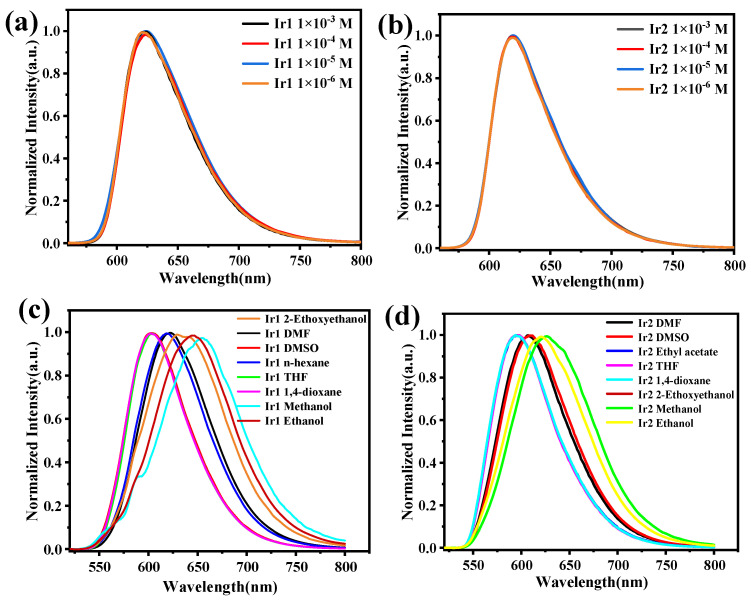
(**a**,**b**) Emission spectra of Ir1 and Ir2 at different concentrations in dichloromethane at room temperature, (**c**,**d**) Emission spectra of Ir1 and Ir2 in different solvents at the concentration of 1.0 × 10^−5^ M.

Next, the absorption and emission spectra of Ir1 and Ir2 in CH_2_Cl_2_ were studied at room temperature, as shown in Figure 2, and the photophysical data have been summarized in Table 1. The absorption spectra of both complexes showed a similar profile, and the intense absorption peak below 300 nm can be ascribed to π-π* transition of the main ligand; the relatively weak peak between 322 to 400 nm may be ascribed to a mixture of the ^1^MLCT (singlet metal-to-ligand charge transfer) and ^3^MLCT (triplet metal-to-ligand charge transfer). Notably, a weak absorption peak existed at a range of 472 to 565 nm, which may be assigned to ^3^MLCT [28]. Both Ir1 and Ir2 exhibited bright-red emission in CH_2_Cl_2_ via the naked eye when excited by 365 nm, as depicted in the insert in Figure 2b. The maximum phosphorescent peaks of Ir1 and Ir2 were at 625 and 620 nm with the full-width emission at half maximum of 66 and 59 nm, respectively. The lifetimes of Ir1 and Ir2 in the degassed CH_2_Cl_2_ are 140 and 130 ns, respectively, which showed typical exponential decay, and the relatively short lifetime is beneficial to the device’s performance. Impressively, the two complexes exhibited a high-phosphorescent quantum efficiency of 0.32 and 0.35, respectively. Then, the phosphorescent behaviors of both complexes in degassed CH_2_Cl_2_, at various concentrations in the range of 1.0 × 10^−3^ to 1.0 × 10^−6^ M, were studied, as shown in Figure 3a,b. The emission spectra showed a maximum phosphorescent peak at 625 nm for Ir1 and 620 nm for Ir2, respectively. The similar profile indicated that there existed almost no or very weak intermolecular interactions between the adjacent molecules in the solution. The low-temperature (77 K) phosphorescent spectra were then investigated, as shown in Figure 2d; Ir1 and Ir2 showed obvious blue-shift compared with the spectra at room temperature, peaking at 607 and 596 nm with a shoulder peak at 653 and 649 nm, and the rigidochromic shift for Ir1 and Ir2 are 18 and 24 nm, respectively. Furthermore, according to the highest energy vibronic sub-band of emission spectra at 77 K, the triplet energy (T_1_) can be calculated to be 2.05 eV for Ir1 and 2.08 eV for Ir2. The phosphorescent properties of Ir1 and Ir2 at solid-states were then investigated. Both of them showed bright-red emission via the naked eye when excited by 365 nm UV light (Figure S2), and the maximum phosphorescent peaks were at 700 and 674 nm, respectively.

Additionally, the phosphorescent behaviors of Ir1 and Ir2 in various degassed solvents (1,4-dioxane, 2-ethoxyethanol, DMF, DMSO, ethanol, methanol, n-hexane, and THF) at the same concentration were carried out and depicted in (Figure 3c,d). The maximum phosphorescent peak of both Ir1 and Ir2 changed significantly together with the solvents possessing different polarity. Taking Ir1 as an example, the maximum emission peak of Ir1 in 1,4-dioxane and methanol is 602 and 656 nm, respectively, which exhibited a large shift about 54 nm. The distinct shift indicated that the phosphorescent emission may have resulted from a mixture of LC excited-state and ^3^MLCT [29,30,31].

To understand the mechanism of photophysical properties of Ir1 and Ir2 in depth, theoretical calculations were then performed via B3LYP and time-dependent density functional theory (TD-DFT) as shown in Figure S2 and Table S1. The highest occupied molecular orbital (HOMO) and the lowest unoccupied molecular orbital (LUMO) energy levels of Ir1 and Ir2 are −5.32/−2.08 eV and −5.48/−2.13 eV, respectively. From the optimized steric configuration, we can see that Ir1 and Ir2 adopted a twisted-octahedral configuration. The frontier molecular orbitals (FMO) suggested that the HOMO and LUMO of both compounds are located on the main ligand and Ir atom, without the auxiliary ligands. The HOMO-1 of Ir1 and the HOMO-1, HOMO+2 of Ir2 are located at the main ligands and Ir atom. The triplet excited-states of both compounds can be ascribed to a mixture of MLCT transition and LLCT transition. Although, the auxiliary ligands did not involve FMO construction, which may be a good explanation of the similar photophysical properties of the two complexes.

### 2.3. Cyclic Voltammograms and TGA Analysis

**Figure 4:**
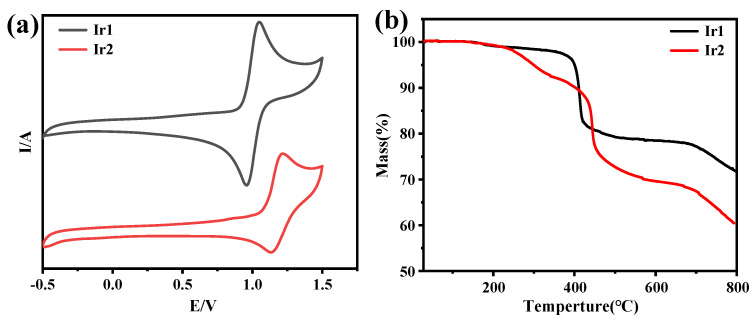
(**a**) The cyclic voltammograms of Ir1 and Ir2 under a scan rate of 100 mV/s in CH_3_CN, (**b**) TGA curves of Ir1 and Ir2.

Furthermore, the cyclic voltammetry was then carried out in acetonitrile to assess the electrochemical properties of Ir1 and Ir2. As shown in Figure 4a, both of them exhibited reversible oxidation wave, and the oxidation potential is determined to be 0.92 and 1.05 V. The Ir-centered oxidation may be responsible for the positive oxidation potential. On the basis of cyclic voltammetry, the energy level of the HOMO and LUMO of Ir1 and Ir2 can be calculated to be −5.73/−3.74 and −5.86/−3.86 eV, respectively.

As we know, thermostability of EML materials in OLEDs based on vacuum evaporation is a very important factor. The thermogravimetric analysis was carried out under argon. The results showed that the temperatures of weight loss at about 5% for Ir1 and Ir2 are at 401 and 301 °C, respectively, suggesting that both compounds possessed excellent thermostability under argon.

### 2.4. Electroluminescent Devices

To investigate the electroluminescence (EL) performances, OLEDs based on Ir1 and Ir2 were carefully fabricated adopting the structures of ITO/MoO_3_ (3 nm)/TCTA (4,4′,4′′-tris(carbazol-9-yl)triphenylamine, 40 nm)/Bepp_2_ (bis [2-(2-pyridinyl)phenolato]berylliuM):4 wt% Ir (20 nm)/TPBi (1,3,5-tris(1-phenyl-1H-benzimidazol-2-yl)benzene, 50 nm)/LiF (1 nm)/Al (100 nm) as shown in Figure S3. Among them, ITO, MoO_3_, TCTA, and TPBi acted as an anode, hole-injection layer, hole-transport layer, and electron-transfer layer, respectively. Bepp_2_, a high-efficiency fluorescent material, acted as the host of phosphors Ir1 and Ir2, and was doped with the EML. The energy level of triplet-excited states for Bepp_2_ is 2.6 eV, higher than Ir1 and Ir2, which can realize an effective Forster energy transfer between the host material and the iridium(III) complexes. To overall assess the device’s performances, a doping concentration of 4% of weight was adopted. The energy levels of the red OLEDs were depicted in Figure S3. On the basis of the energy levels of the devices, the energy level HOMO/LUMO of Ir1 and Ir2 were both within those of the Bepp_2_ host material. Therefore, the excellent carrier capturing in the host-guest mixture system may be responsible for the dominant EL mechanism [32]. The performances of the red devices based on the two complexes were shown in Table 1 and summarized in Table 2.

**Figure 5:**
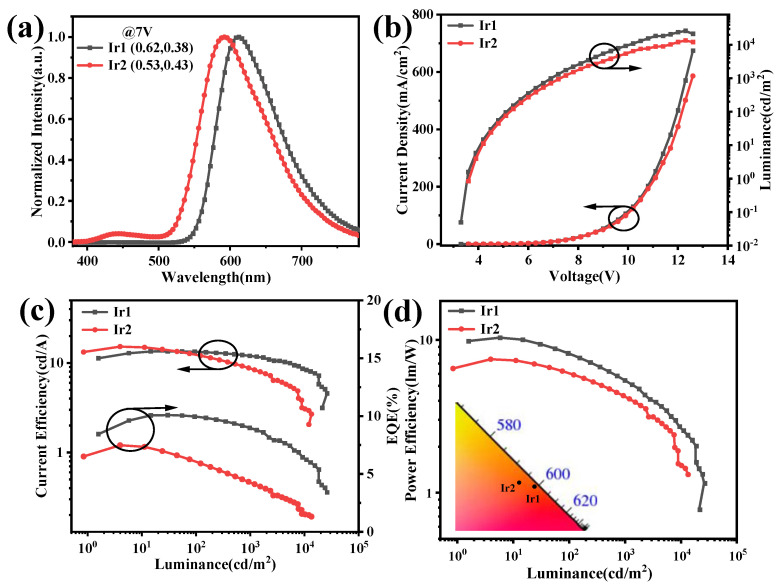
(**a**) The normalized EL spectra, (**b**) The J-V-L curves, (**c**) CE-L-EQE curves, and (**d**) PE-L curves of the red OLEDs based on Ir1 and Ir2 with the doping concentration of 4% in Bepp_2_.

The turn-on voltages at 1 cd/m^2^ of the device based on Ir1 and Ir2 were at 3.5 and 3.7 V, respectively, and these low values indicated a poor injection barrier between electron-transfer layers to the emissive layers. Notably, the normalized EL spectra of Ir1 and Ir2 exhibited obvious blue-shift, compared with those of in CH_2_Cl_2_, peaking at 612 and 591 nm, respectively, which may be attributed to sensitivity within the surroundings of the triplet-excited states of these two complexes, and the host material Bepp_2_ may be an important influence on the triplet-excited states, which coincides with the obvious solvatochromism behaviors in different solvents. The 1931 Commission Internationale de L’Eclairage (CIE) coordinates of two devices are (0.61, 0.37) and (0.51, 0.43), respectively, which are corresponding to the red range. The L-V-J curves of the red devices were depicted in Figure 5b. The key parameters of devices were listed in Table 2. The maximum luminance of Ir1 and Ir2 are 25,970 and 13,450 cd/m^2^, respectively. The maximum current efficiency (CE), power efficiency (PE), and external quantum efficiency (EQE) of the red devices based on Ir1 and Ir2 are 13.47/15.22 cd/A, 10.35/12.26 lm/W, and 10.08/7.48%, respectively.

## 3. Conclusions

In summary, two novel neutral-phosphorescent iridium(III) complexes (Ir1 and Ir2) have been successfully designed and synthesized with high yields and good solubility. Both complexes exhibited enhanced red emission in the solid and solution. The devices based on Ir1 and Ir2 were rationally fabricated by using Bepp2 as the host material with the doping concentration of 4%. The performances of these devices showed some differences, and the peak CE, PE, and EQE of the red devices based on Ir1 and Ir2 are 13.47/15.22 cd/A, 10.35/12.26 lm/W, and 10.08/7.48%, respectively. Owing to sensitivity within the surroundings of the triplet-excited states of these two complexes, EL spectra exhibited a degree of blue-shift compared with those of in CH_2_Cl_2_. Next, we will select more appropriate host materials and device structures to improve the red-EL quality of the OLEDs. At last, we believe that our work will be of great significance for the development of novel red-emissive iridium complexes in the future.

## Figures and Tables

**Table 1:** Photophysical data of Ir1 and Ir2.

Complexes	Emission in CH_2_Cl_2_			E_g_ [eV] ^a^	E_onset_^ox^ [eV]	T_1_ [eV] ^b^
	λ_em_ [nm]	Τ [μs]	Φ_PL_			
Ir1	625	0.14	0.32	2.23	0.92	2.05
Ir2	620	0.13	0.35	2.23	1.05	2.08

^a^ E_g_ was estimated from absorption onset from UV-visible spectra. ^b^ T_1_ = 1240/λ_77K_.

**Table 2:** EL data for the red OLEDs based on Ir1 and Ir2.

Complex	X %	λλ_EL_/ nm	CIE(x,y)	V_ON_/ V	L_max_/ cd·m^−2^	CE_max_/ cd·A^−1^	PE_max_/ lm·W^−1^	EQE/ %
Ir1	4	612	(0.61, 0.37)	3.5	25,970	13.47	10.35	10.08
Ir2	4	591	(0.51, 0.43)	3.7	13,450	15.22	12.26	7.48

## Data Availability

Raw data are available from the authors upon request.

## Supplementary Materials

The following supporting information can be downloaded at: https://www.mdpi.com/article/10.3390/molecules28124865/s1, Figure S1: The phosphorescent spectra of Ir1 and Ir2 in solid states. Figure S2: The optimized structures, distributions of molecular orbitals of Ir1 and Ir2. Figure S3: Schematic diagram of device structure (**a**) of the red OLEDs and chemical structures (**b**) of the materials involved in the prepared devices. Table S1: The theoretical calculations of molecular orbital for the two complexes. Refs. [33,34,35,36,37] are cited in Supplementary Materials.

## Sample Availability

Samples of the compounds are not available from the authors.
