# Supplementary material for: Two Novel Neutral Cyclometalated Iridium(III) Complexes Based on 10,11,12,13-Tetrahydrodibenzo[a,c]phenazine for Efficient Red Electroluminescence
Source: Molecules. 2023 Jun 20;28(12):4865. doi: 10.3390/molecules28124865 (PMC10303947; doi:10.3390/molecules28124865)
Supplement: Supplementary file 1 [file molecules-28-04865-s001.zip › molecules-2432092-supplementary.pdf]

Supporting Information

Two novel neutral cyclometalated-iridium(III) complexes based on 10,11,12,13-tetrahydrodibenzo[a,c]phenazine for efficient red electroluminescence

Yuzhen Yang[1], Han Zhao[1], Weiqiao Zhou[1], Qin Zeng[1], Zihao Zhang[1], Junjie Jiang[1], Yongyang Gong[1], Yanqin Miao*, Song Guo[1,*], and Yuanli Liu[1,*]

[1] Guangxi Key Laboratory of Optical and Electronic Materials and Devices, College of Materials Science and Engineering, Guilin University of Technology, Guilin 541004, China; yangyuzhen812@163.com(Y.Y.); 1020200156@glut.edu.cn (H.Z.); wangyi123zhou@163.com(W.Z.); zqinnn@163.com(Q.Z.); 1020190104@glut.edu.cn (Z.Z.); jiejiangjjj0513@163.com(J.J.); yygong@glut.edu.cn (Y.G.);

[2] MOE Key Laboratory of Interface Science and Engineering in Advanced Materials, Taiyuan University of Technology, Taiyuan 030024, PR China.

* Correspondence: bobingjin@glut.edu.cn (S.G.), lyuanli@glut.edu.cn (Y.L.).

1. General Experimental Information

The NMR spectra were measured with a Bruker spectrometer at room temperature. Mass spectra were obtained on Bruker matrix-assisted laser desorption/ionization time-of-flight mass pectrometer (MALDI-TOF-MASS). The UV-vis absorption spectrum was recorded on a Lambda 750 spectrometer (PerkinElmer, America). Emission spectra and lifetimes were measured on a FluoroMax-4 fluorescence spectrophotometer (Horiba, Japan). Photographs were taken by Canon 90D camera (Canon, Japan). The absolute quantum yields of the complexes was determined through an absolute method by employing an integrating sphere. Cyclic voltammetry curves were accomplished in CH_2Cl_2 with a three-electrode cell configuration consisting of platinum working and counter electrodes and a $Ag/AgNO_3$ (0.01 M in CH_3CN) reference electrode under N_2 atmosphere at room temperature. Tetra-*n*-butylammonium hexafluorophosphate (0.1 M in CH_2Cl_2) was used as the supporting electrolyte. The redox potentials were recorded at a scan rate of 100 mV/s and are reported with reference to the ferrocene/ferrocenium (Fc/Fc^+) redox couple.

All OLEDs were fabricated on pre-patterned ITO glass substrates with a sheet resistance of 15 Ω sq-1. ITO-coated glass substrates were cleaned by ultrasonic cleaning in deionized water, acetone and isopropyl alcohol for 20 min in order, then treated with ultraviolet-ozone for 15 min. After this, the ITO substrates were led into a deposition chamber, and the OLEDs were fabricated through a vacuum evaporation deposition process under a pressure below 5×10-4 Pa. The deposition rates and film thicknesses were monitored by calibrated crystal quartz sensors. The deposition rates for the organic materials, LiF, MoO3 and Al were 1~2, 0.3, 0.5, and 3~6 Å s-1, respectively, in particular, the evaporation rate of EMLs is about 0.1 Å s-1. All the devices had an active emissive area of 3 mm×3 mm, which are defined by the overlap between the ITO anode and Al cathode. The current J-V-L characteristics were performed simultaneously by using a computer-controlled source meter (Keithley 2400) integrated with a BM-7A luminance meter test system. The EL spectra were recorded by a PR655 spectrometer. The EQE was calculated from the current density-voltage-luminance curves and EL spectral data.

2. Calculation method

The ground-state geometrical configuration was optimized by density functional theory (DFT) with B3LYP functional. Based on the optimized ground state molecular structure, the time-dependent DFT (TDDFT) approach associated with the polarized con-tinuum model (PCM) in dichloromethane media was carried out to obtain the vertical excitation energies of triplet states (Tn). The calculation was performed using the Gaussian 16 B.01 suite of programs. The SDD basis set was used to treat the iridium atom, whereas the 6-31G* basis set was used to treat all other atoms. The contours of the highest occupied molecular orbital (HOMO) and lowest unoccupied molecular orbital (LUMO) were plotted by Multiwfn 3.8 soft. [S5-6]

3. Synthesis of cyclometalating ligand and iridium(III) Complexes

Synthetic routes of **Ir1** and **Ir2** are described in **Figure 1**.

Synthesis of main ligand: 10,11,12,13-tetrahydrodibenzo[a,c]phenazine: A mixture of cis-1,2-cyclohexanediamine (6.00 g) and 9,10-phenanthraquinone (10.76 g) (n:n=1:1) were added to a 250 mL round-bottom flask by using ethanol as the solvent. Then the system was heated to 110 °C for 48 h. After completion of the reaction, the product was cooled and recrystallized, yielding a white solid. ^{1}H NMR (500 MHz, $CDCl_3$, δ)： 8.28 (dd, J=1.0 Hz, 2.0 Hz, 2H), 8.00 (d, J=7.95 Hz, 2H), 7.52 (dt, J=8.3 Hz, 1.2 Hz, 2H), 7.38 (t, J=7.35 Hz, 2H), 2.93-2.85 (m, 2H), 2.51-2.48 (m, 2H), 1.93-1.91 (m, 2H), 1.66-1.57 (m, 2H), 1.50-1.43 (m, 2H). 13C NMR (125 MHz, $CDCl_3$, δ）:153.18, 133.26, 131.51, 131.10, 128.39, 126.04, 123.30, 58.76, 33.61, 25.74.

Synthesis of Iridium(III) Complex:

The *μ*-dichloro bridged Ir(III) dimer complex 2 was synthesized according to the published literature. [S1-4] $IrCl_3 \cdot 3H_2O$ (1.23 g, 3.5 mmol) and the ligand 1 (0.7 g, 7.0 mmol) were added into a mixture of 2-ethoxyethanol and deionized water (60 mL, 3:1 v/v). Then the system was heated to 110 °C under N_2 atmosphere for 15 h. After the system was cooled, the orange solid was obtained by filtering the solution and then washed with deionized water and methanol. Next, the solid was dried using a vacuum

drying oven.

The synthesis of **Ir1** and **Ir2** are according to the previously reported method. [S1-4]

Ir1/Ir2: μ-dichloro bridged Ir(III) dimer complex 2 (0.3 g, 0.19 mmol), 1.0 g K_2CO_3, acetylacetone and picolinic acid (38 mg /47 mg, 0.38 mmol) were added into a mixture of degassed dichloromethane (40 mL), the system was stirred at room temperature for 5 h under nitrogen atmosphere. Then the solvent was removed by a rotary evaporator. The crude product was purified by column chromatography using dichloromethane/methanol as eluent.

Ir1: red powder (70% yield). ^{1}H NMR (500 MHz, CD_2Cl_2, δ): 9.20 (d, J=7.45 Hz, 2H), 8.53 (d, J=7.75 Hz, 2H), 7.96 (d, J=10 Hz, 2H), 7.77-7.70 (m, 4H), 7.00 (t, J=7.65 Hz, 2H), 6.42 (d, J=6.85 Hz, 1H), 3.61-3.54 (m, 2H), 3.43 (t, J=6.4 Hz, 4H), 2.17-2.13 (m, 3H), 1.63 (s, 5H), 1.51 (m, 5H), 1.24 (s, 5H). 13C NMR (125 MHz, $CDCl_3$, δ）:185.71, 156.00, 152.59, 151.92, 143.75, 141.45, 139.52, 132.50, 132.50, 131.62, 130.93, 129.28, 128.75, 127.11, 124.83, 123.09, 114.55, 101.38, 33.19, 31.60, 30.53, 28.38, 23.15, 22.36, 14.12. MALDI-TOF-MS (m/z): calcd for C45H37IrN4O2, 858.03; found, 858.74.

Ir2: red powder (72% yield). ^{1}H NMR (500 MHz, $CDCl_3$, δ): 9.21 (d, J=8.0 Hz, 2H), 8.60 (s, 1H), 8.51 (s, 1H), 8.25 (s, 1H), 8.09 (s, 1H), 8.00 (s, 1H), 7.82-7.69 (m, 5H), 7.50 (s, 1H), 7.21-7.04 (m, 3H), 6.71 (s, 1H), 6.32 (s, 1H), 5.30 (s, 2H), 3.80-3.72 (m, 1H), 3.33 (s, 4H), 2.97-2.80 (m, 2H), 2.09-2.00 (m, 4H), 1.26 (s, 4H). 13C NMR (125 MHz, $CDCl_3$, δ）:156.50, 153.75, 152.55, 150.75, 147.61, 140.34, 138.02, 132.35, 132.05, 131.32, 130.21, 129.77, 129.09, 127.64, 127.61, 127.23, 125.12, 122.97, 115.75, 114.87, 29.34, 27.23, 23.10, 22.80, 21.81. MALDI-TOF-MS (m/z): calcd for $C_{46}H_{32}Cl_2F_6IrN_4O_2$, 881.03; found, 881.86.

References

[S1] Guo, S.; Guo, C. X.; Lu, Z.; Du, L. L.; Gao, M.; Liu, S. J.; Liu, Y. L.; Zhao, Q. Crystals. 2021, 11, 1190.

[S2] Sun, H. B.; Liu, S. J.; Lin, W. P.; Zhang, K. Y.; Lv, W.; Huang, X.; Huo, F. W.; Yang, H. R.; Jenkins, G.; Zhao, Q.; Huang, W.. Nat. Commun. 2014, 5, 3601.

[S3] Tao, P.; Li, W. L.; Zhang, J.; Guo, S.; Zhao, Q.; Wang, H.; Wei, B.; Liu, S. J.; Zhou, X. H.; Yu, Q.; Xu, B. S.; Huang, W. Adv. Funct. Mater. 2016, 26, 881.

[S4] Nonoyama, M. Bull. Chem. Soc. Jpn. 1974, 47, 767.

[S5] Frisch, M.J.; Trucks, G.W.; Schlegel, H.B.; Scuseria, G.E.; Robb, M.A.; Cheeseman, J.R.; Scalmani, G.; Barone, V.; Petersson, G.A.; Nakatsuji, H.; Li, X.; Caricato, M.; Marenich, A.V.; Bloino, J.; Janesko, B.G.; Gomperts, R.; Mennucci, B.; Hratchian, H.P.; Ortiz, J.V.; Izmaylov, A.F.; Sonnenberg, J.L.; Williams, Ding, F.; Lipparini, F.; Egidi, F.; Goings, J.; Peng, B.; Petrone, A.; Henderson, T.; Ranasinghe, D.; Zakrzewski, V.G.; Gao, J.; Rega, N.; Zheng, G.; Liang, W.; Hada, M.; Ehara, M.; Toyota, K.; Fukuda, R.; Hasegawa, J.; Ishida, M.; Nakajima, T.; Honda, Y.; Kitao, O.; Nakai, H.; Vreven, Y.; Throssell, K.; Montgomery J, J.A.; Peralta, J.E.; Ogliaro, F.; Bearpark, M.J.; Heyd, J.J.; Brothers, E.M.; Kudin, K.N.; Staroverov, V.N.; Keith, T.A.; Kobayashi, R.; Normand, J.; Raghavachari, K.; Rendell, A.P.; Burant, J.C.; Iyengar, S.S.; Tomasi, J.; Cossi, M.; Millam, J.M.; Klene, M.; Adamo, C.; Cammi, R.; Ochterski, J.W.; Martin, R.L.; Morokuma, K.; Farkas, O.; Foresman, J.B.; Fox, D.J.; Wallingford. CT. 2016.

[S6] Lu, T.; Chen F. W. Multiwfn. J. Comput. Chem. 2012, 33, 580-592.

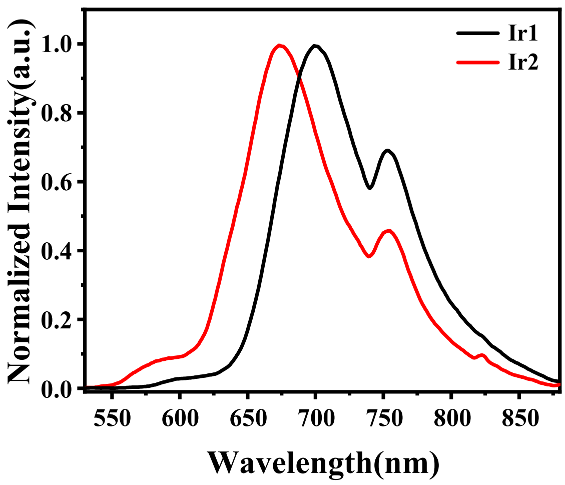

Figure S1. The phosphorescent spectra of Ir1 and Ir2 in solid states.

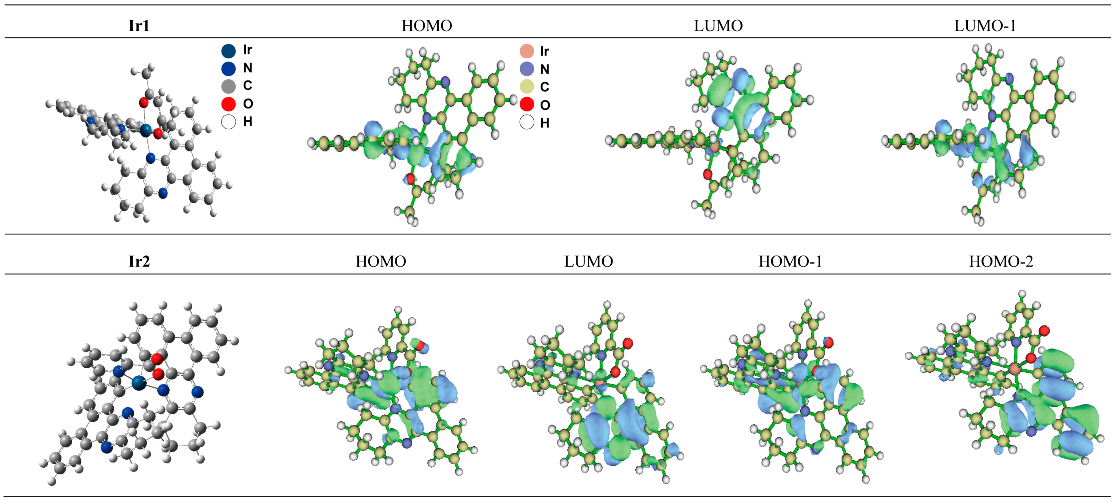

Figure S2. The optimized structures, distributions of molecular orbitals of Ir1 and Ir2.

Table S1 The theoretical calculations of molecular orbital for the two complexes.

Complexes	State	HOMO (eV)	LUMO (eV)	Configuration	Character
				HOMO→LUMO, 75.1%	MLCT
**Ir1**	T_1	-5.32	-2.08	HOMO-1→ LUMO, 10.7%	MLCT/LLCT
				HOMO → LUMO, 71.3%	MLCT/LLCT
**Ir2**	T_1	-5.48	-2.13	HOMO-1→ LUMO, 10.9%	MLCT/LLCT
				HOMO-2 → LUMO, 7.7%	LLCT

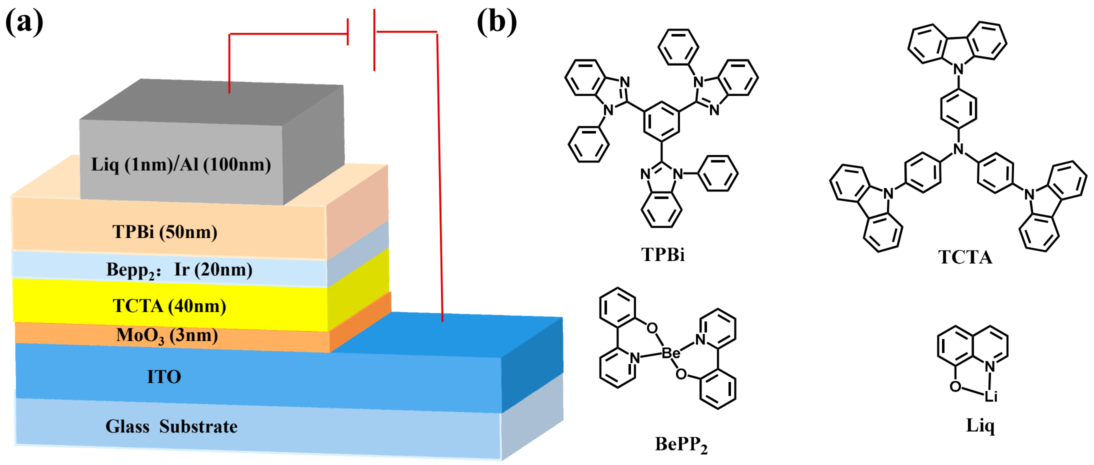

Figure S3. Schematic diagram of device structure (a) of the red OLEDs and chemical structures (b) of the materials involved in the prepared devices.

NMR and MS Spectra

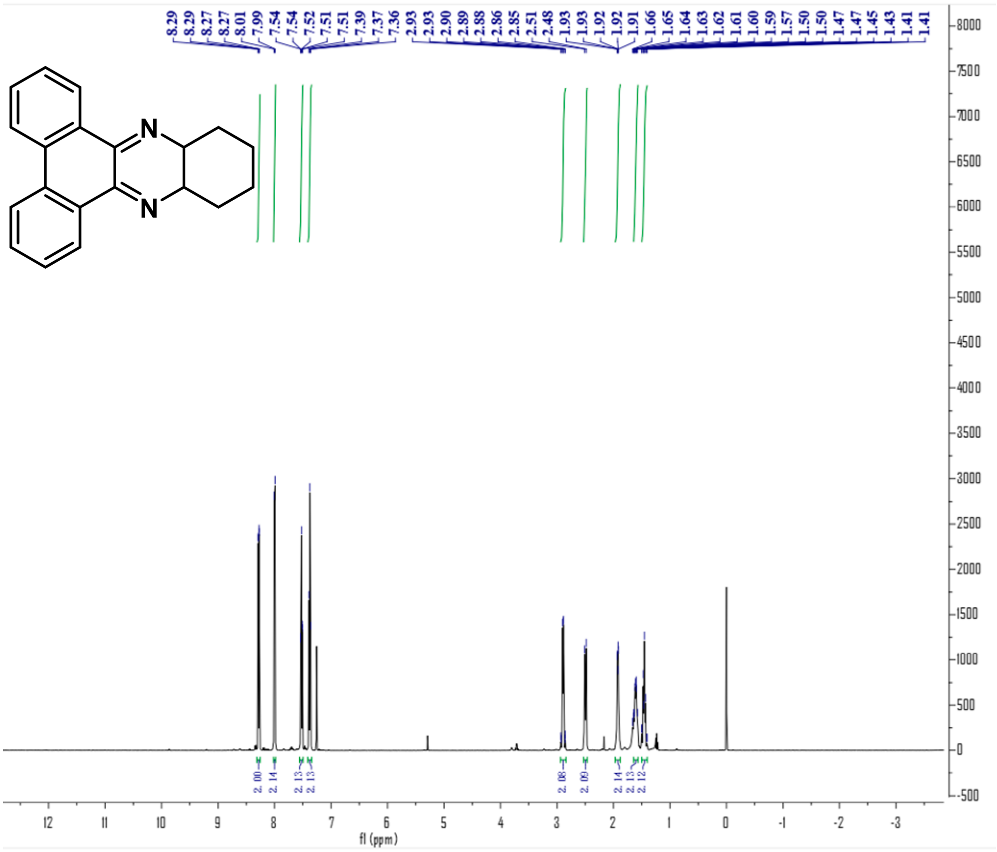

The ^{1}H NMR of main ligand.

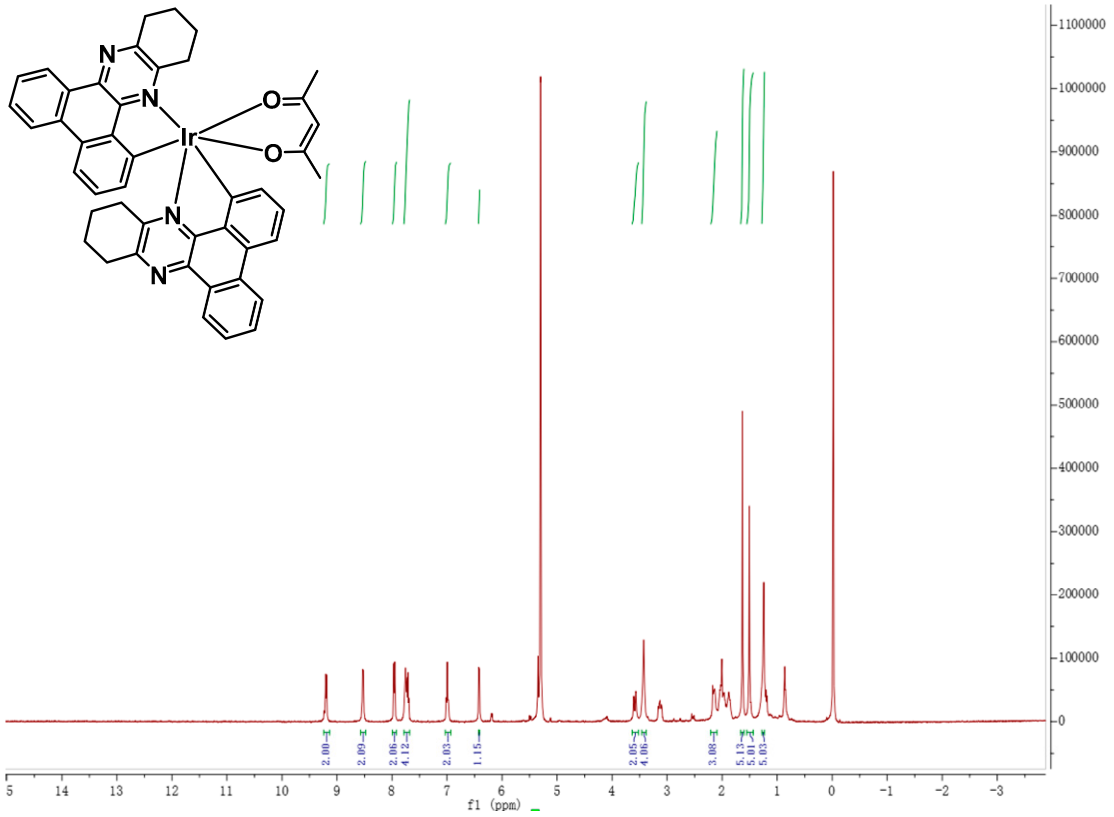

The ^{1}H NMR of complex Ir1.

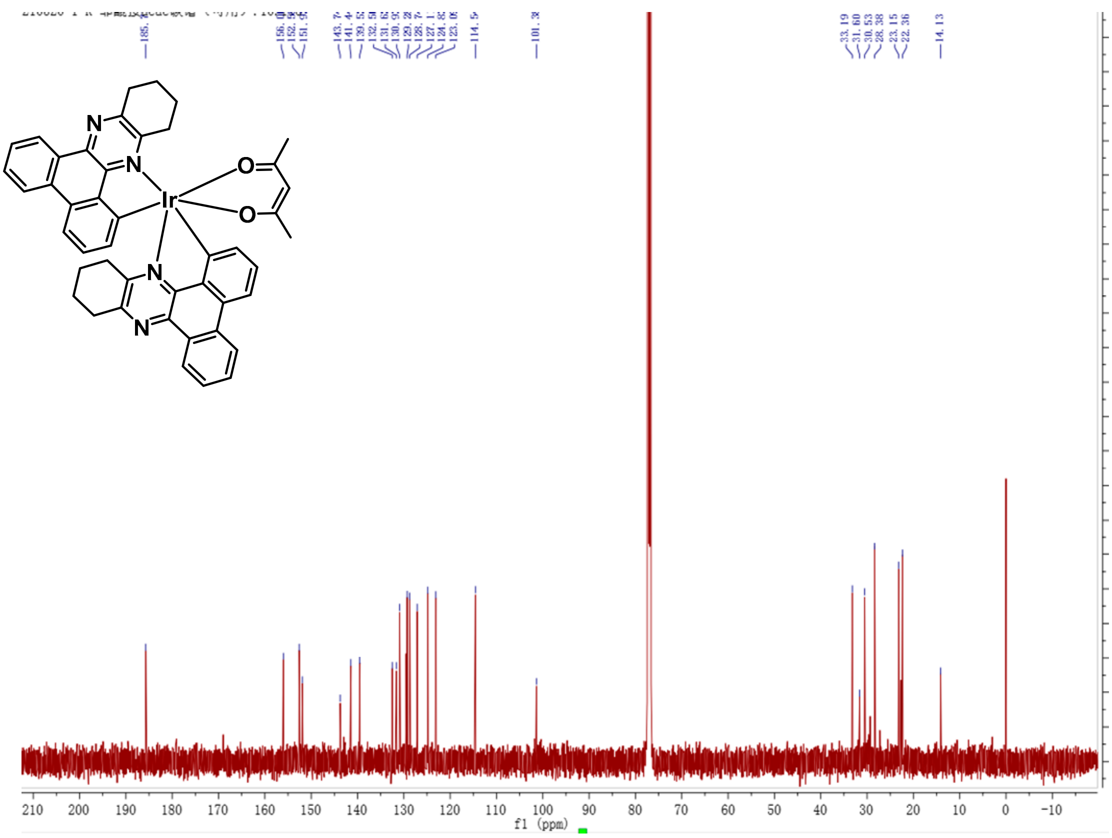

The ^{13}C NMR of complex Ir1.

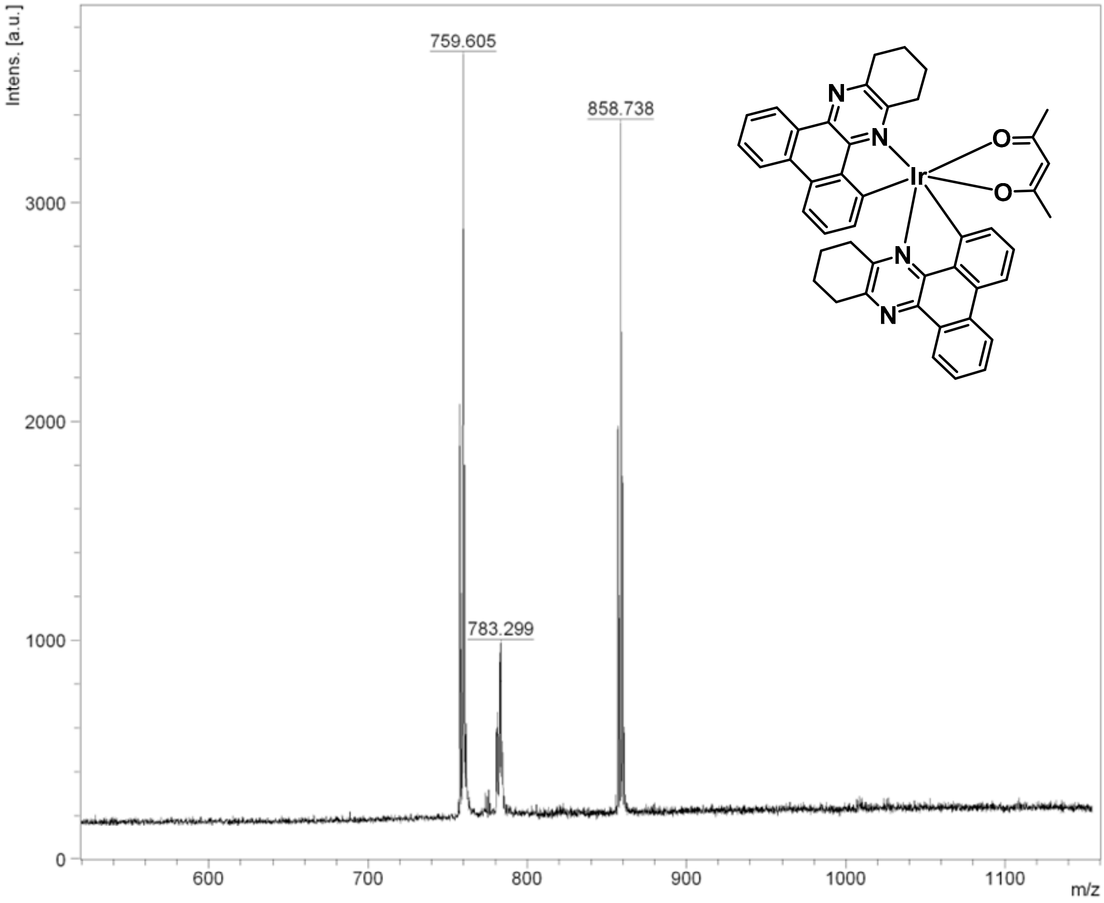

The MALDI-TOF-MASS of complex Ir1.

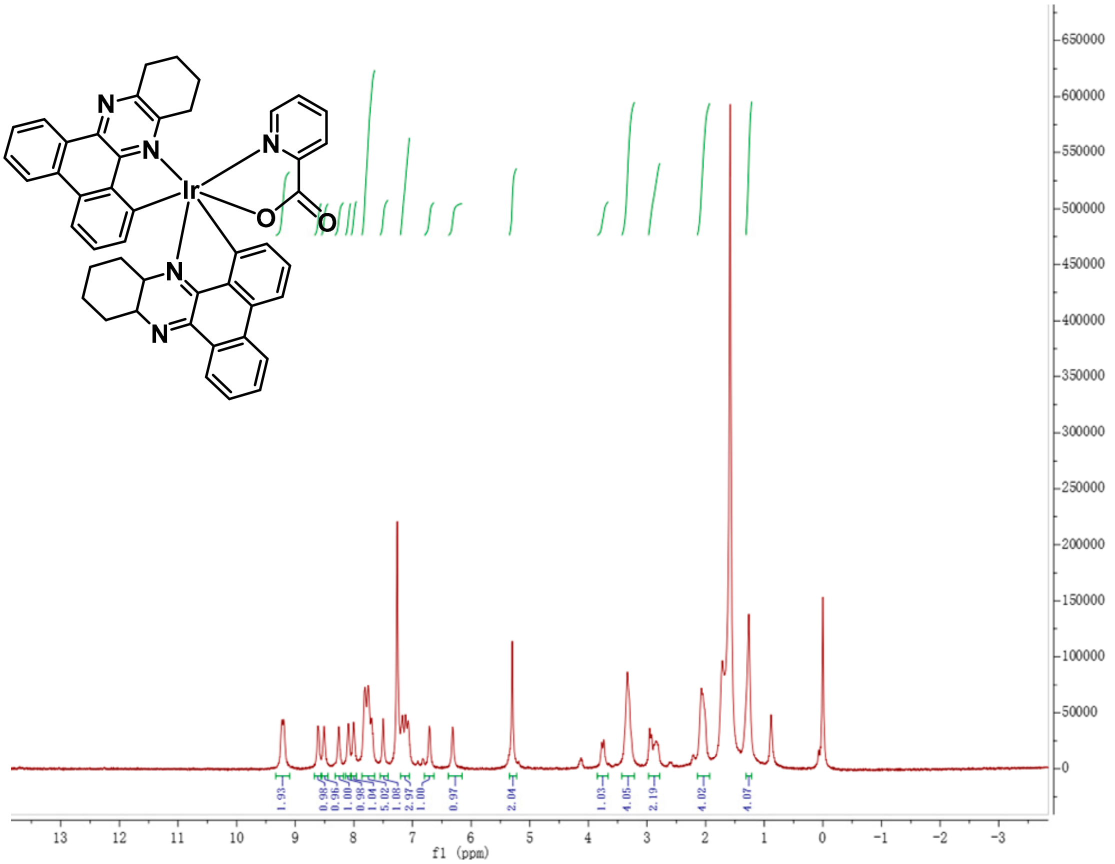

The ^{1}H NMR of complex Ir2.

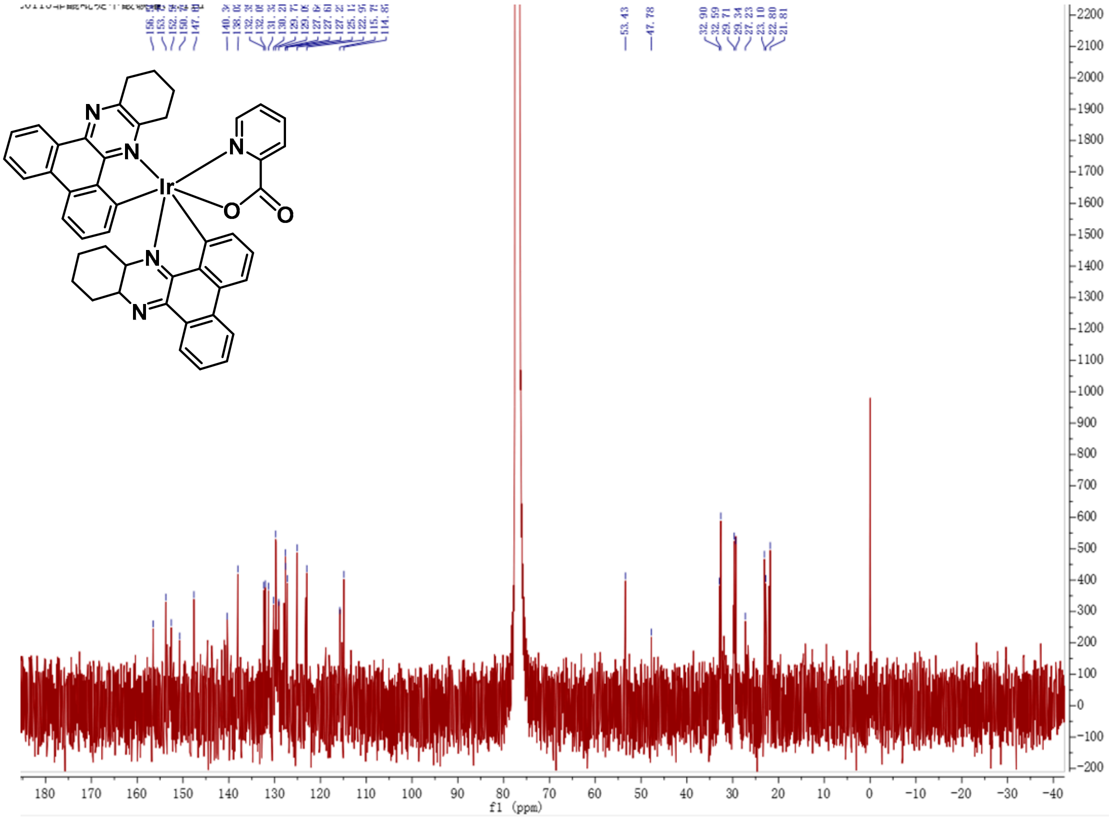

The ^{13}C NMR of complex Ir2.

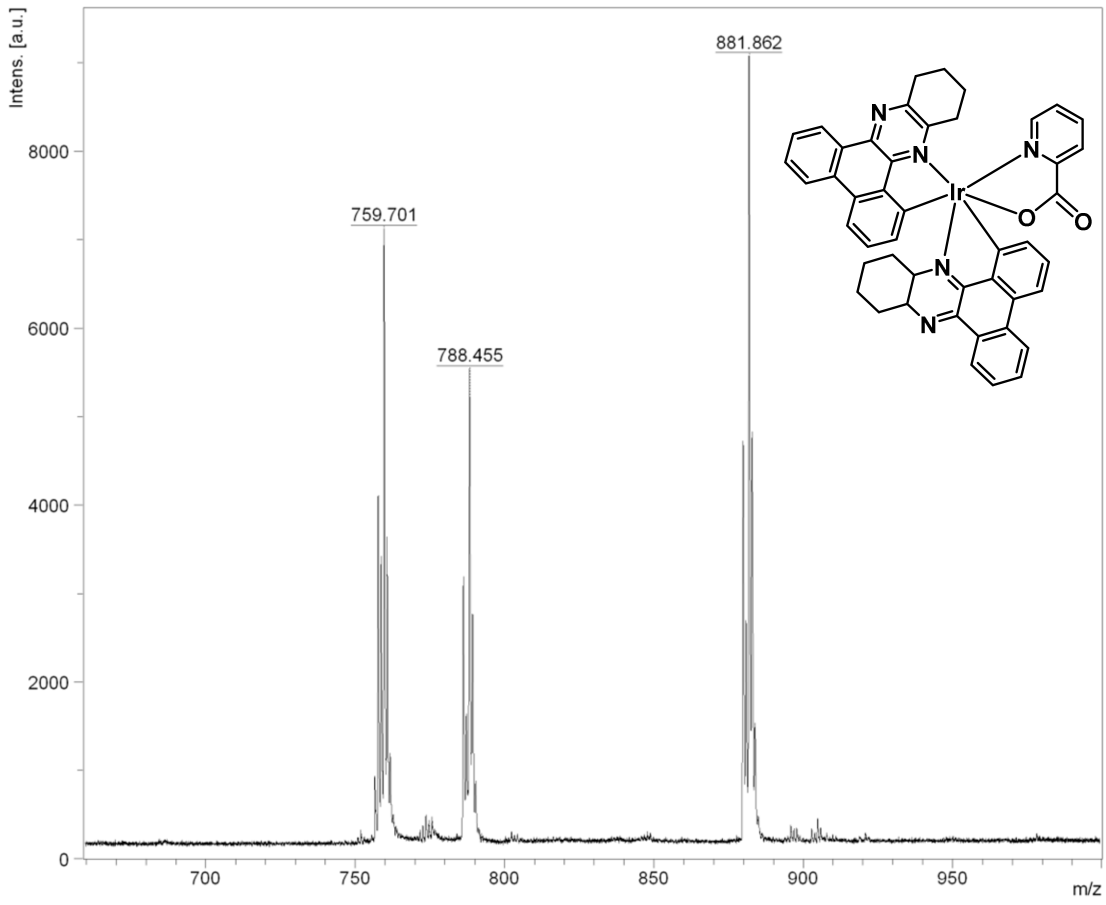

The MALDI-TOF-MASS of complex Ir2.
